# Occurrence of *cagA*^+^*vacA s1a m1 i1 Helicobacter pylori* in farm animals in Egypt and ability to survive in experimentally contaminated UHT milk

**DOI:** 10.1038/s41598-018-32671-0

**Published:** 2018-09-24

**Authors:** Mahmoud Elhariri, Dalia Hamza, Rehab Elhelw, Eman Hamza

**Affiliations:** 1 Department of Microbiology and Immunology, Faculty of Veterinary Medicine, Cairo University, Giza, Egypt; 2 Department of Zoonoses, Faculty of Veterinary Medicine, Cairo University, Giza, Egypt

## Abstract

Cases of human gastric cancer due to *Helicobacter pylori* have been reported worldwide and animals might act as a reservoir of infection in certain circumstances. The recent few decades showed a rapid decline in the incidence of gastric cancer, which was mainly due to the decrease in *H*. *pylori* infection. The aims of the present study were to determine the prevalence of *H*. *pylori* among livestock and investigate whether the animal isolates can be transmitted through contaminated milk causing gastric infection. Feces and milk samples were collected from apparently healthy cows, buffaloes, and sheep, and were examined by nested PCR and genotyping. The PCR positive samples were further subjected to bacterial culture followed by partial 16s sequencing of the isolates. Twenty-nine percent of the animals showed the presence of *H*. *pylori*, mainly the virulent *cagA*^+^*vacA*^+^*s1a m1 i1* genotype, which is known to be associated with serious diseases in humans. The spiral viable culturable form (SVCF) of this strain was inoculated into UHT (ultra-high temperature) milk and remained viable for up to 10 days at 4 °C. Increasing period of storage and or temperature led to a decrease in the number of the SVCF and occurrence of the coccoid viable non-culturable form (CVNCF). The infectivity of the survived forms was determined by feeding healthy groups of laboratory mice with the contaminated UHT milk containing SVCF or CVNCF for 40 days. The gastric mucosa of the two mice groups showed similar levels of *H*. *pylori* load. This highlights that *H*. *pylori* can persist in contaminated milk by entering a non-culturable state, which can induce gastric infection.

## Introduction

*Helicobacter pylori* is the most important etiological agent of chronic gastritis and peptic ulcer^1,2^. Infection with *H*. *pylori* is common worldwide with an estimated prevalence of 70% in developing countries and 30% to 40% in industrialized countries^3^. In Egypt, studies have shown a high prevalence of *H*. *pylori* infection among apparently healthy adult individuals^4–6^, school children^7^, and newborns^8^. The majority of colonized patients do not show any symptoms, while long-term carriage of this pathogen significantly increases the risk of developing gastric cancer^9,10^. The clinical outcome of infection with *H*. *pylori* depends on the bacterial survival and virulence factors as well as host factors. *Helicobacter pylori* is a gram-negative bacterium which has been characterized for a long time in terms of spiral viable culturable form (SVCF)^11^. However, the existence of a coccoid viable non-culturable form (CVNCF) was demonstrated several years ago^12,13^. The transformation from the default SVCF to CVNCF can occur under adverse environmental conditions^14^ to facilitate long-term bacterial survival. The CVNCF cannot be detected by the ordinary culture method^15^, but rather by direct electron microscopy^13^ and molecular techniques^12^. Furthermore, one of the unique features of *H*. *pylori* is its ability to persist in the acidic environment through urease production and thus facilitate gastric epithelium colonization^16^.

A number of virulence factors were found to determine the pathogenicity of *H*. *pylori*. Of these, the vacuolating cytotoxin (vacA) is of particular concern as it contributes to the longevity of infection^17^ and has a pleiotropic effect on host cells, including vacuolization cytotoxicity and apoptosis^18^. *VacA* gene is polymorphic in four variable regions, the most characterized are the signal sequence (s), the mid (m) and the intermediate (i)^19^. Each of these regions show allelic diversity, the s is designated as s1 (s1a, s1b, and s1c) and s2, the m is categorized to m1 and m2, and the i-region consists of i1, i2, and i3^19^. This variation is linked to specific clinical outcomes, for example, *H*. *pylori* strains that carry s1-m1 or -m2 are more virulent than those with s2 alleles, whereas the i-region is thought to determine the carcinogenic ability of the strains^19^. Another important virulence factor is the cytotoxin-associated gene product (cagA) which is encoded on the cag pathogenicity island (PAI). Although all *H*. *pylori* strains possess vacA, only some of them are cagA positive^20^. Studies indicate that the carriage of cagA is related to virulence as well as to the development of human gastric cancer^21^.

Little is known about the exact reservoir of *H*. *pylori*; some studies have suggested animals as natural hosts. This refers to the presence of *H*. *pylori* in the gastric mucosa of different animal species^22^ with mild or absence of an inflammatory response^23^. A body of evidence suggests that *H*. *pylori* can survive for long periods in food of animal origin^23–27^. However, it is unlikely that *H*. *pylori* survives the pasteurization process, milk can be contaminated post-pasteurization^28^. Interestingly, *H*. *pylori* was found to survive longer in the UHT milk than in the pasteurized milk, which was explained by the presence of competitive microbiota in the latter that can influence the survival of *H*. *pylori*^28^. The UHT milk is widely used globally, because of their safety and longer shelf life over the raw and pasteurized milk^29^. Therefore, the aims of the present study were to examine the occurrence of *H*. *pylori* among healthy livestock in Egypt, characterize the bacterial virulence genotypes, to investigate the length of survival of the animal isolates in contaminated UHT milk as well as their ability to be transmitted through milk via the oral route and causing gastric infection in healthy laboratory mice groups.

## Results

### Occurrence of *Helicobacter pylori* in fecal and milk samples from apparently healthy farm animals using PCR

**Figure 1:**
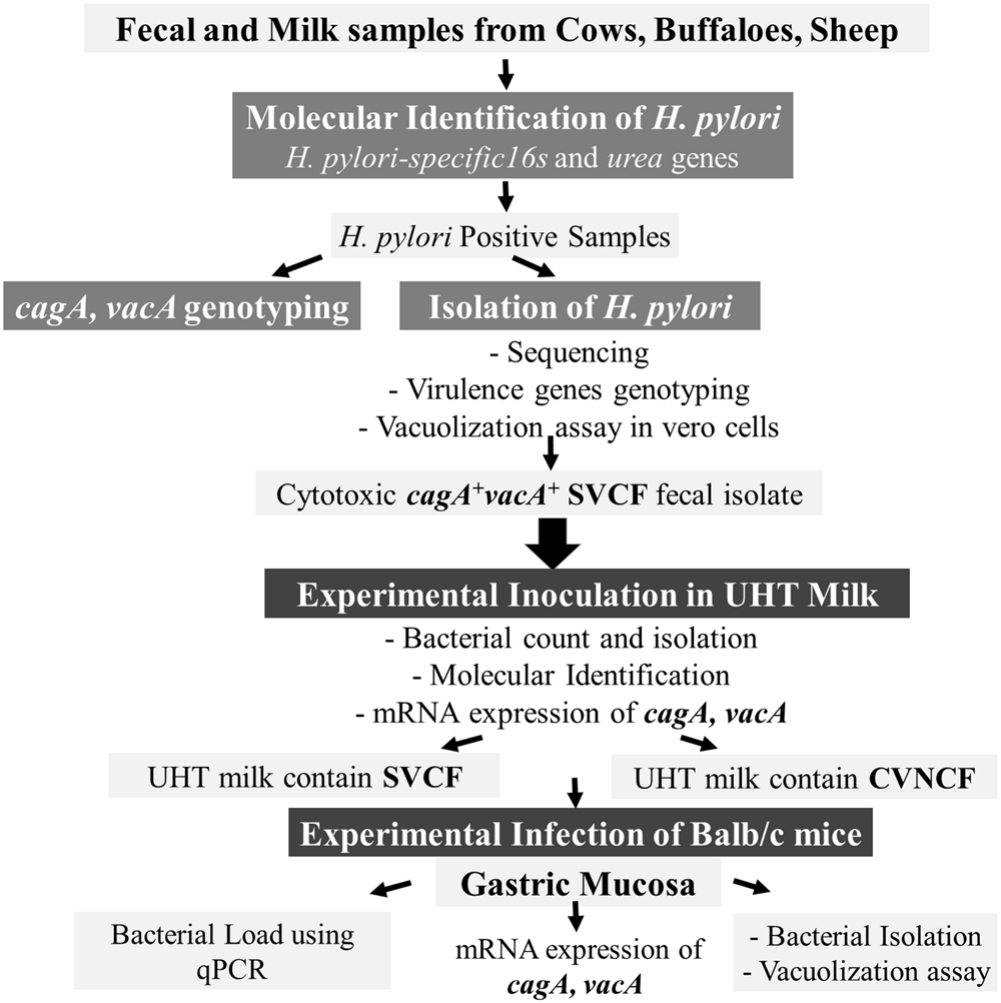
Schematic flow chart of the study design. Fecal and milk samples were collected from apparently healthy livestock and were subjected to PCR targeting *H*. *pylori*-specific *16s* and *ureA* genes. The PCR positive samples were further subjected to genotyping of the virulence genes *cagA* and *vacA* and to bacteriological culture. The bacterial isolates were examined for *cagA* and *vacA* genotypes and for cytotoxicity using vacuolization assay. The fecal isolate with predominant cytotoxic genotype *cagA*^+^ *vacA s1a m1 i1* was inoculated in UHT milk and its survivability under different temperatures was determined. The UHT milk that contains the survived *H*. *pylori* strains were fed to healthy experimental mice groups for 40 days. The gastric mucosa was collected following scarification of the mice groups and was examined by real-time/quantitative polymerase chain reaction (qPCR) to estimate the load of infection. The gastric mucosa was further subjected to bacteriological culture and the mRNA expression of *cagA* and *vacA* was detected by reverse transcription PCR (RT-PCR).

The experimental design of the present study is illustrated in Fig. 1. Since *H*. *Pylori* can be present in a non-culturable form^11–14^, nested PCR that targets *H*. *pylori*-specific 16s rRNA and conventional PCR for *ureA* genes (Fig. S1a, S1b) were used (Table 1). It is important to note that all positive samples showed the presence of both genes. No amplification was detected in the negative control. Table 2 reveals a significantly higher prevalence of *H*. *pylori* 51.42% (36/70) in cows compared to 15% (9/60) in buffaloes and 16% (8/50) in sheep. However, 22.9% (16/70) of cows showed the presence of *H*. *pylori* in feces alone, 7.1% (5/70) in milk alone, and 21.4% (15/70) in both milk and feces. This shows that a significantly higher number of cows showed the presence of *H*. *pylori* in feces alone than in milk. Unlike in cows, the 9 positive buffaloes showed the presence of *H*. *pylori* in feces alone (n = 4) and in both milk and feces (n = 5), but not in milk alone. Similar to buffaloes, in the 8 positive sheep, *H*. *pylori* was found in feces alone (n = 5) or in both milk and feces (n = 3).

**Table 1:** Sequences of the primers used in PCR and the amplification conditions.

Genes	Primers: Sequences (5′-3′)	PCR product size (bp) ^(References)^	PCR conditions
Helicobacter *pylori*-specific-16s rRNA	HP1-R: CTGGAGAGACTAAGCCCTCC HP2-F: ATTACTGACGCTGATTGTGC HP3-F: AGGATGAAGGTTTAAGGATT	109^54^	Nested PCR In the first amplification round, Hp1 and Hp3 primers were used to amplify template DNA. In the second amplification round, Hp1 and Hp2 primers were used to amplify 1 μl PCR product from the first amplification. The first and second amplifications 92 °C, 2 min; 60 °C, 2 min; 72 °C, 2 min; final step at 72 °C, 4 min (35 cycles).
*Urea*	HPU1-F: GCCAATGGTAAATTAGTT HPU2-R: CTCCTTAATTGTTTTTAC	411^57^	95 °C, 5 min; 94 °C, 1 min; 45 °C, 1 min; 72 °C, 1 min; final step at 72 °C, 5 min (35 cycles).
*vacA s1a*	F: GTCAGCATCACACCGCAAC R: CTGCTTGAATGCGCCAAAC	190^58^	94 °C, 1 min; 58 °C, 1 min; 72 °C, 1.5 min (35 cycles).
*s1b*	F: AGCGCCATACCGCAAGAG R: CTGCTTGAATGCGCCAAAC	187^58^	
*s2*	F: GCTAACACGCCAAATGATCC R: CTGCTTGAATGCGCCAAAC	199^58^	
*m1*	F: GGTCAAAATGCGGTCATGG R: CCATTGGTACCTGTAGAAAC	290^58^	
*m2*	F: GGAGCCCCAGGAAACATTG R: CATAACTAGCGCCTTGCAC	352^58^	
*i1*	F1: GTTGGGATTGGGGGAATGCCG C1R: TTAATTTAACGCTGTTTGAAG	426^59^	
*i2*	F1: GTTGGGATTGGGGGAATGCCG C2R: GATCAACGCTCTGATTTGA	432^59^	
*cagA-PAI*	F: AGGGATAACAGGCAAGCTTTTGAC R: TG CAAAAGATTGTTTGGCAGA	352^59^	94 °C, 1 min; 58 °C, 1 min; 72 °C, 1.5 min (35 cycles).
*cagA-empty site*	F: CCAAATACATTTTGGTAAATAAAC R: CTCTTTTTGTGCCTTTGATTGAA	550^60^	94 °C, 1 min; 58 °C, 1 min; 72 °C, 1.5 min (30 cycles).
*Helicobacter genus-specific-16s rRNA*	C97-F: GCTATGACGGGTATCC C05-R: ACTTCACCCCAGTCG CTG	1200^62^	94 °C, 1 min; 55 °C, 2.5 min; 72 °C, 3 min (35 cycles).
*SSA*	F: TGGCGTGTCTATTGACAGCGAGC R: CCTGCTGGGCATACTTCACCATG	303^66^	95 °C, 5 min; 40 cycles of 95 °C, 30s, 65 °C, 30s, and 72 °C, 30s.

**Table 2:** Occurrence of *Helicobacter pylori* among livestock using PCR targeting *H. pylori*-specific *16s* and *ureA* genes.

Species	Total No tested	Animals positive for *H. pylori*-specific *16s rRNA and ureA* genes							
		Feces		Milk		Both		Total positive animals	
		No	%	No	%	No	%	No	%
Cows	70	16	22.9*^§^	5	7.1	15	21.4*	36	51.4*
Buffaloes	60	4	8.3	0	0	5	8.3	9	15
Sheep	50	5	10	0	0	3	6	8	16
Total	180	25	13.8	5	2.8	23	12.8	53	29.4

* Indicates significant difference from the other species of animals with a p value of 0.00253 using a chi - square test. ^§^ Indicates significant difference from milk within the same animal with a p value of 0.03 using McNemar test.

### Predominant expression of *cagA*^+^*vacA*^+^*s1* genotype combination among the *Helicobacter pylori* PCR positive strains

The samples positive for *H*. *pylori* 16s and *ureA* genes (n = 76) were subjected to conventional PCR targeting the two virulence genes *cagA* (s, m, and i regions) and *vacA* (Fig. S2). Out of the 76 examined *H*. *pylori* strains, 58 carried the two genes (*cagA*^+^*vacA*^+^), while 18 contained *vacA* gene alone (*cagA*^−^*vacA*^+^) (Table 3). All strains showed allelic diversity in *vacA* gene with a predominant expression of *s1* over *s2* alleles. Among the 58 *cagA*^+^*vacA*^+^ strains, 50 were *s1* positive and 8 *s2* positive. Similarly, of the 18 *cagA*^−^*vacA*^+^ strains, 13 carried *s1* allele and 5 harbored *s2* allele. Furthermore, the major genotype combinations detected in all samples were represented by *cagA*^+^*vacA*^+^*s1a m1 i1* and *cagA*^+^*vacA*^+^*s1a m2 i1*. Interestingly, there were no differences in the genotype of the virulence genes between strains from different species of animals (Table 3) or strains from different type of samples (data not shown).

**Table 3:** Genotype combinations of *Helicobacter pylori in* PCR positive samples.

Genotype				No of *H*. *pylori* positive samples (n = 76)			
*CagA*	*VacA*			Cows (n = 51)	Buffaloes (n = 14)	Sheep (n = 11)	Total
	*s*	*M*	*i*				
*cagA* ^+^	***s1a***	***m1***	***i1***	17	4	3	**24**
		***m2***	***i1***	9	4	4	**17**
	***s1b***	***m1***	***i1***	3	2	n	**5**
		***m2***	***i2***	n	4	n	**4**
	*s2*	*m1*	*i2*	3	n	n	3
		*m2*	*i2*	5	n	n	5
*Total cagA* ^+^ *vacA* ^+^				**37**	**14**	**7**	**58**
*cagA* ^*−*^	***s1a***	***m1***	***i1***	2	n	n	**2**
		***m2***	***i1***	3	n	1	**4**
	***s1b***	***m1***	***i1***	4	n	n	**4**
		***m2***	***i2***	n	n	3	**3**
	*s2*	*m1*	*i2*	1	n	n	1
		*m2*	*i2*	4	n	n	4
*Total cagA* ^−^ *vacA* ^+^				**14**	**n**	**4**	**18**

n, means negative.

### Bacterial culture of the PCR positive samples followed by genotyping and sequencing of the isolates

**Figure 2:**
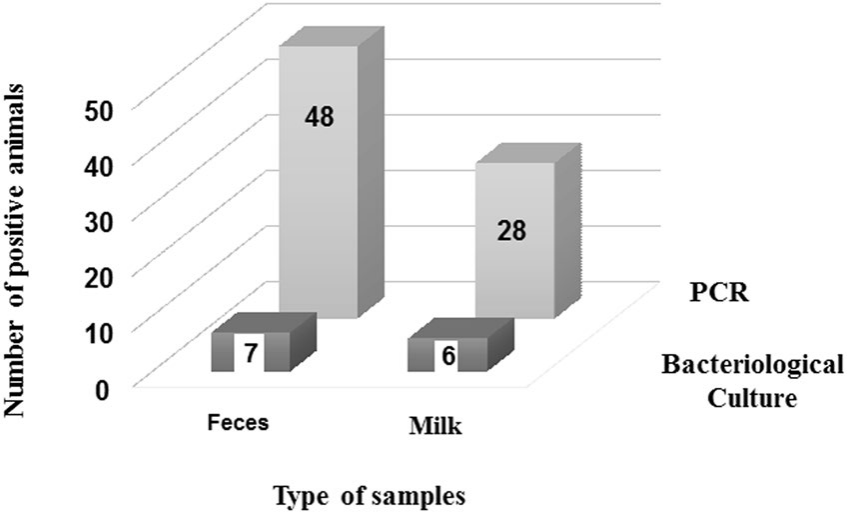
Number of PCR positive samples that contained the spiral viable culturable form of *Helicobacter pylori* as determined by bacteriological culture and identification. Milk and fecal samples from the three species of animals that were positive using PCR were subjected to bacteriological culture and biochemical identification. The results are shown as total numbers of milk samples (Milk) and fecal samples (Feces) that were tested positive in PCR (PCR) and numbers of PCR positive samples that showed positive colonies of *H*. *pylori* (Bacteriological Culture).

The 76 PCR positive genotyped samples, including 48 fecal and 28 milk samples were subjected to bacterial culture, microscopic examination and biochemical identification (Fig. 2). Thirteen (7 fecal and 6 milk) samples were positive (culturable), the isolated bacteria were rod shaped gram negative, reacted positively in urease, catalase and oxidase tests, and were negative to nitrate reduction and glycine utilization tests. This shows the presence of the spiral viable culturable form of *H*. *pylori* (SVCF). The negative control samples represented by PCR negative fecal (n = 2) and milk (n = 2) samples were negative in the bacterial culture, whereas the positive control samples were culturable. In order to confirm the virulence genotype following bacterial culture, the 13 isolates were subjected to conventional PCR targeting the two virulence genes *cagA* (s, m, and i regions) and *vacA*. This revealed that 12 isolates were of the virulence genotype *cagA*^+^*vacA*^+^*s1a m1 i1* and one isolate was of the *cagA*^−^*vacA*^*+*^*s1b m2 i1* genotype.

Since these isolates will be further used in the experimental part of the study, it was necessary to confirm their identity. Accordingly, the obtained 13 isolates were sequenced based on *Helicobacter*-genus specific 16s. All the isolates were confirmed to be *H*. *pylori*, excluding the presence of other *Helicobacter* species. The accession numbers of our isolates in the NCBI gene bank are as follows: cow’s milk (KY463207, KY463208, KY463209), buffalo’s milk (KY463210, KY463211), sheep’s milk (KY463212), cow fecal (KY463213, KY463214, KY463215, KY463216), buffalo fecal (KY463217, KY463218), and sheep fecal (KY463219) isolates.

**Figure 3:**
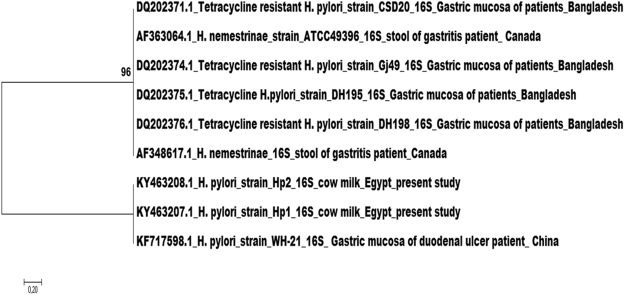
Evolutionary relationship between the strains isolated in the present study and those retrieved from the gene bank based on *Helicobacter* genus-specific 16s. The phylogenetic tree was constructed by using the neighbor-joining method and the evolutionary analysis was conducted in MEGA7. The analysis involved nine nucleotide sequences, two represents the strains of the present study and eight that had 90–100% identity with our sequences. The evolutionary distances were calculated and expressed in the units of the number of base differences per sequence. The numbers on the nodes (shown on the left next to the branches) indicate the number of times (percentage) the species grouped together.

The nucleotide sequences were examined for their genetic relatedness. The Matrix identification plot showed a 99–100% identity between the 13 isolates (data not shown). The evolutionary relation between our strains and those retrieved from the NCBI gene bank was determined by using phylogenetic analysis. Figure 3 shows that our strains are clustered in the same clade with an *H*. *pylori* strain isolated from the gastric mucosa of duodenal ulcer patients in China. Interestingly, our strains shared a common ancestor with a clade of *H*. *pylori* human strains isolated from the gastric mucosa of patients in Bangladesh. Unfortunately, there were no sequences found in the gene bank for *H*. *pylori* isolated from animals.

### Survival of the spiral viable culturable form (SVCF) of *Helicobacter pylori* in artificially contaminated ultra-high temperature (UHT) milk stored at 4 °C for up to 10 days

**Figure 4:**
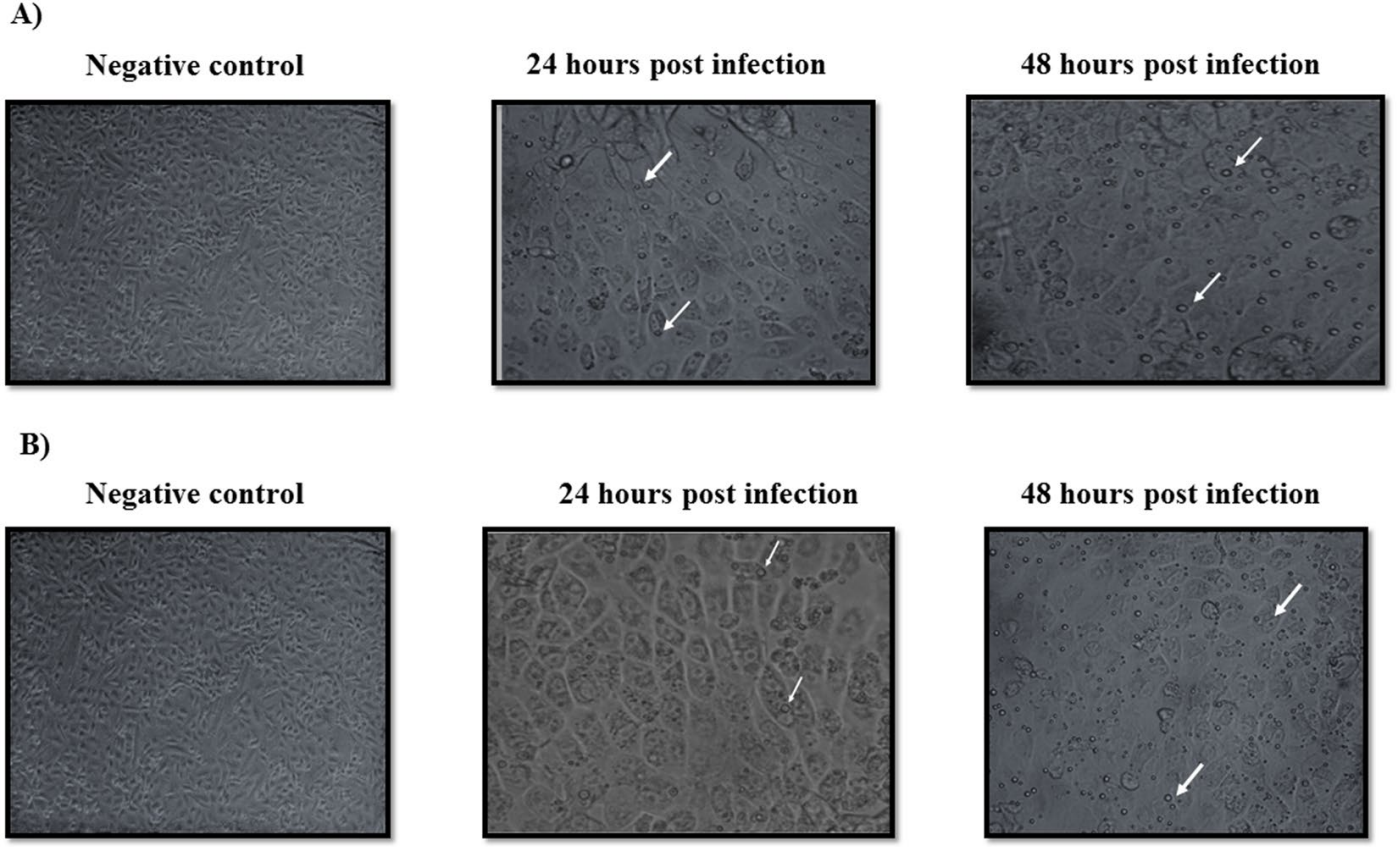
Ability of the *cagA*^+^*vacA s1a m1 i1* strain to induce vacuolization of vero cells. Broth culture filtrate of *H*. *pylori* test strains (**A**) fecal cow isolate before experimental inoculation in pasteurized milk and (**B**) the strain isolated from the experimentally infected mice were added to vero cells and incubated for 24 and 48 hours at 35 °C in the presence of CO_2_. Un-inoculated brucella broth was used as a negative control. Intracellular vacuolization (pointed out with white arrows) was read under inverted microscope with 400x magnification. The densities of the cells shown are 5.5 × 10^6^.

It was of interest to examine whether the SVCF of *H*. *pylori* isolates from feces of animals can survive in contaminated UHT milk. Since not all *H*. *pylori* strains are cytotoxic^15^, the vacuolization activity of the strain used in this experiment was examined and showed formation of intracellular vacuoles in vero cells (Fig. 4A).

**Figure 5:**
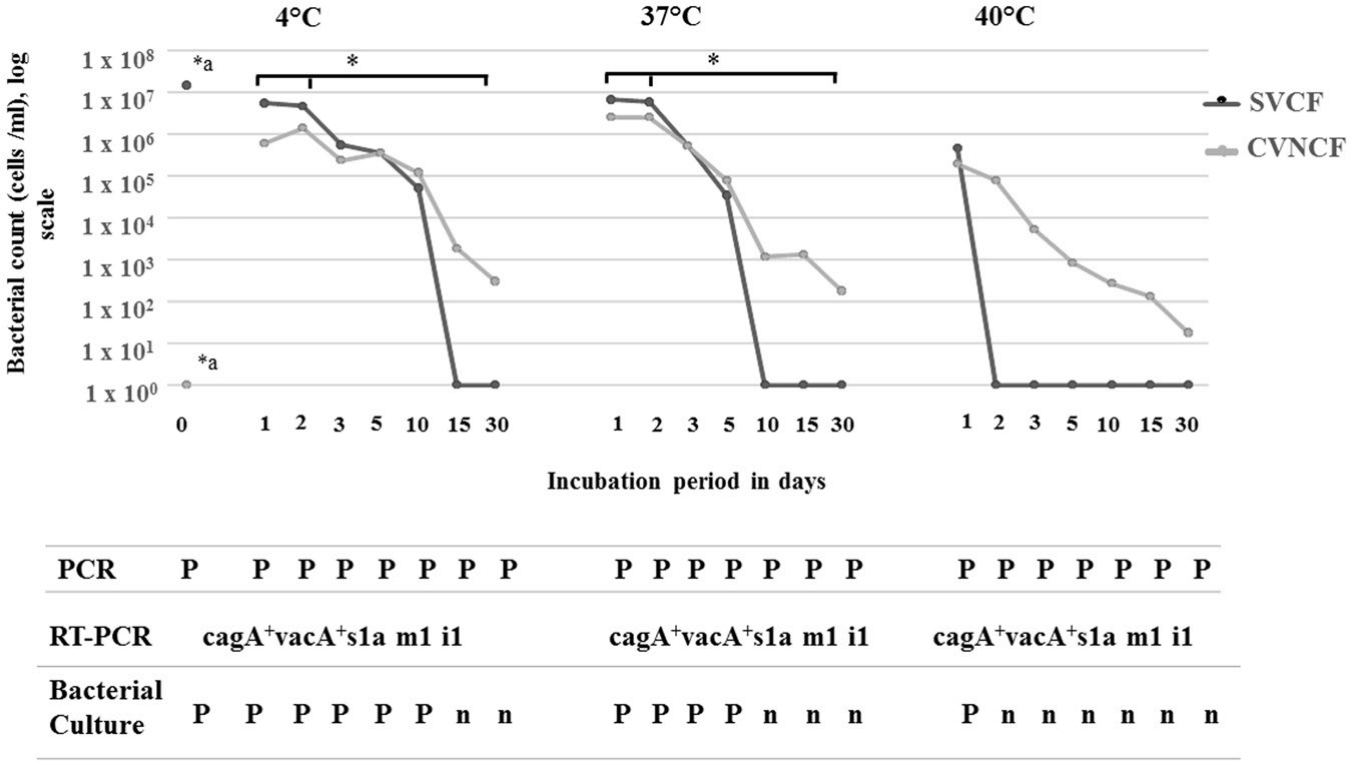
Survival of *Helicobacter pylori* in experimentally contaminated UHT milk. UHT milk was artificially inoculated with cytotoxic spiral viable culturable form (SVCF) of *H*. *pylori* strains (1.5 × 10^7^ cells/ml, 0 hour) of the genotype *cagA*^+^*vacA*^+^*s1a m1 i1* isolated from cow fecal samples. The milk was incubated at 4 °C, 37 °C and 40 °C for a time period from 1 to 30 days. After each time period, the numbers of the spiral viable culturable form (SVCF) and coccoid viable non-culturable form (CVNCF) present in milk were counted using a haemocytometer. *a indicates a significant difference in the bacterial count between 0 hour and all other time points, * with horizontal lines indicates significant differences between day 1 as well as day 2 and the subsequent days (day 3 to 30 days) using Repeated Measures ANOVA. The milk was further subjected to PCR, bacterial culture and reverse transcription PCR (RT-PCR); P indicates positive, n means negative.

The UHT milk was tested before the experimental inoculation by PCR and showed the absence of *H*. *pylori* and other *Helicobacter* species (Fig. S3). The change in the number of the SVCF was examined following inoculation in UHT milk and incubation at different temperature and for different time points. Repeated Measures ANOVA revealed a significant effect of the storage time (p = 0.01) as well as the interaction between temperature and storage time (p = 0.01) on the number of the SVCF, whereas the temperature alone did not show a statistically significant effect (p = 0.09). Figure 5 demonstrates the change in the number and form of SVCF of *H*. *pylori* over time following inoculation in UHT milk. Compared to the initial number inoculated (1.5 × 10^7^ cells/ml, day 0), there was a significant decrease in the SVCF after one-day incubation at 4 °C (5.6 × 10^6^ cells/ml) as well as at 37 °C (6.8 × 10^6^ cells/ml). This remained almost stable by day two storage at 4 °C (4.8 × 10^6^ cells/ml) and at 37 °C (6 × 10^6^ cells/ml). On day three, there was a significant decrease in the SVCF at 4 °C (5.6 × 10^5^ cells/ml) and at 37 °C (5.4 × 10^5^ cells/ml). Storage for five to ten days, the decrease in the SVCF was faster at 37 °C (five days, 3.5 × 10^4^ cells/ml; ten days, 0) than at 4 °C (five days, 3.6 × 10^5^ cells/ml; ten days, 5.2 × 10^4^ cells/ml). Furthermore, the SVCF was not detectable after storage for 15 and 30 days at 4 °C as well as at 37 °C. Unlike storage at 4 °C and at 37 °C, the SVCF was only detectable at one-day incubation at 40 °C (4.7 × 10^5^ cells/ml).

Strikingly, the decrease in the SVCF was associated with a noticeable occurrence of the coccoid form following incubation at the three temperatures, 4 °C, 37 °C and 40 °C (Fig. 5). The coccoid form was even detectable at 30 days incubation at 4 °C (3 × 10^2^ cells/ml), 37 °C (1.8 × 10^2^ cells/ml), and 40 °C (1.8 × 10^1^ cells/ml), after the disappearance of the spiral form (Fig. 5). The viability of the coccoid and spiral forms were confirmed morphologically under the microscope and using urease activity, suggesting that the coccoid form might be the coccoid viable non-culturable form of *H*. *pylori* (CVNCF). This observation was further verified by subjecting the UHT milk sample containing the spiral form and that containing the coccoid form to bacterial culture and PCR. However, both UHT milk samples were *H*. *pylori*-specific *16s* and *ureA* PCR positive, only the UHT milk harbored the SVCF was positive in bacterial culture (Fig. 5). Interestingly, RNA from all PCR positive strains express *H*. *pylori*-specific 16s and was of the same genotype *cagA*^+^*vacA*^+^*s1a m1 i1* as the inoculated strain (Figs 5, S4A–E, lanes 3–5), excluding contamination with strains other than the inoculated one that might have been occurring during the experiment.

### Capability of the coccoid viable non-culturable form (CVNCF) occurred in UHT contaminated milk to induce gastric infection in Balb/C mice

The infectivity of the SVCF and CVNCF of *H*. *pylori* that survived in the contaminated UHT milk was investigated using mouse model. Prior to the experimental infection, all mice groups were shown to be Helicobacter-free (Fig. S3) and the contaminated UHT milk given to the mice was verified to contain either SVCF alone, or CVNCF alone.

**Figure 6:**
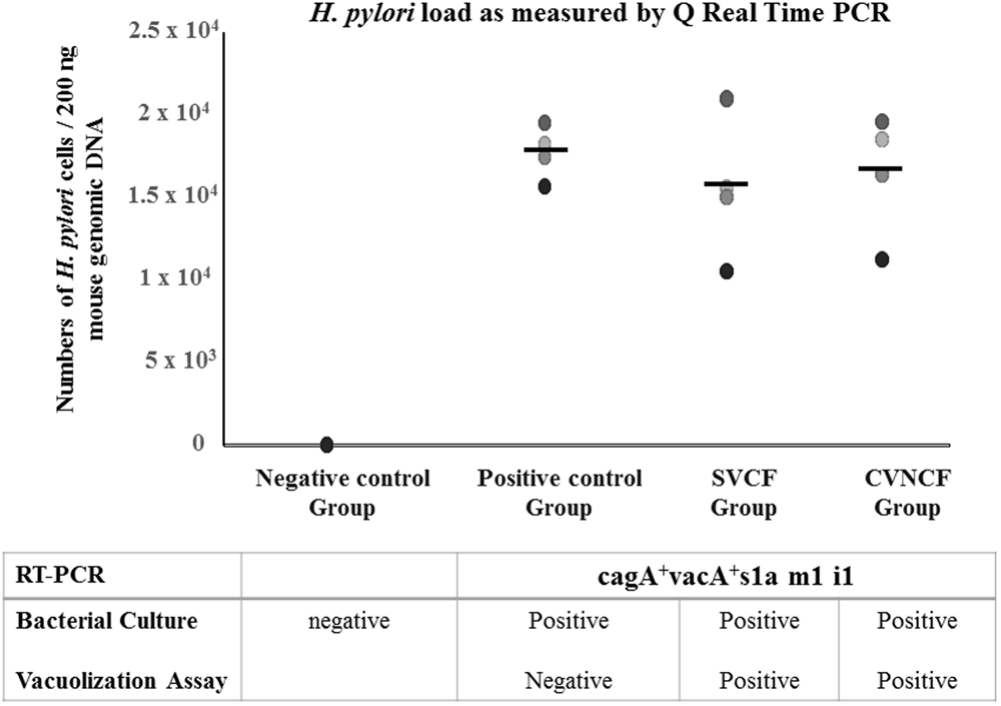
Load of *Helicobacter pylori* in gastric mucosa of the experimentally infected groups of balb c/mice as measured by quantitative real-time polymerase chain reaction (qPCR). Four groups of mice were fed orally uninfected UHT milk (*Negative control Group*) or UHT milk containing SS1 reference strain (*Positive control Group*), spiral viable culturable form (*SVCF Group*) or coccoid viable non-culturable form of *H*. *pylori* (*CVNCF Group*). Each dot represents an individual mouse, the middle horizontal lines indicate mean values. Bacterial load was measured in the gastric mucosa using qPCR and the results are shown as number of bacterial cells per 200 ng of mouse genomic DNA. Homogenates of the gastric mucosa were also examined for mRNA expression of *H*. *pylori* virulence genes using RT-PCR and were further subjected to bacterial culture.

Four groups of mice were given orally UHT milk containing SVCF (*SVCF group*), CVNCF (*CVNCF group*), SS1 reference strain (*Positive control group*) or uninfected milk (*Negative control group*). Figure 6 illustrates a high *H*. *pylori* load in gastric mucosa of the *SVCF* (mean 1.6 × 10^4^; range 1.1 × 10^4^ − 2.1 × 10^4^) and the *CVNCF* (1.6 × 10^4^; 1.1 × 10^4^ − 2 × 10^4^) *Groups* similar to that in the *Positive control Group* (1.8 × 10^4^; 1.6 × 10^4^ − 2 × 10^4^) as estimated by real time/quantitative PCR (qPCR). Additionally, the gastric mucosa of the *Negative control group* that received uninfected milk showed the absence of *H*. *pylori* DNA. Furthermore, *H*. *pylori* strains that colonized the gastric mucosa of the *SVCF* and the *CVNCF* groups express mRNA of *H*. *pylori*-specific 16s, *cagA* and *vacA s1a m1 i1* like those present in the UHT milk given to mice, whereas the gastric mucosa samples from the *Negative control group* were negative to the *H*. *pylori*-specific 16s rRNA (Fig. S4A–E, lanes 1–2, 6–7).

Bacterial culture of the gastric mucosa of the *SVCF group* confirmed the presence of the culturable form of *H*. *pylori*. However, the *CVNCF Group* received UHT milk containing non-culturable form of *H*. *pylori*, the gastric mucosa were positive in the bacterial culture and biochemical identification (Fig. 6). Both strains of *H*. *pylori* isolated from the *SVCF* and *CVNCF Groups* were cytotoxic as shown in the vacuolization assay (Fig. 4B). Conversely, the gastric mucosa from the *Negative control Group* was negative in the bacterial culture.

## Discussion

The prevalence of *H*. *pylori* in Egypt is high^2^, particularly in children^7,8^. We demonstrated recently a high sero-prevalence of *H*. *pylori* in humans having contact with animals^6^. Additionally, other studies reported that people that have direct contact with farm animals such as abattoir workers^30^ and shepherds^31,32^ have a high prevalence of *H*. *pylori* infection, suggesting a role of animals in the transmission cycle. In the present study, fecal and milk samples from livestock were examined by nested PCR followed by genotyping of the virulence genes *vacA* and *cagA*. The prevalence of *H*. *pylori* was 29.4%, which is similar to that reported in other studies (19, 21, 27%)^6,33,34^. Comparing the occurrence of *H*. *pylori* based on the species of animals demonstrated that cows had higher prevalence (51.3%) than sheep (16%) and buffaloes (15%). This is consistent with other studies^24,33–36^ that showed infection of the three animal species with *H*. *pylori*, although the hosts with the highest prevalence have been always controversial, reflecting that the natural hosts might vary according to geographical location^37^. Furthermore, an overall high prevalence of the animals positive for *H*. *pylori* showed the presence of the bacteria only in the feces (47%, 25/53), while the presence of *H*. *pylori* in milk was mainly associated with its presence in feces (43%, 23/53) than in milk alone (9%, 5/53). This indicates that feces are the main shedding site, which agrees with the findings^22,34^ that *H*. *pylori* colonnizes the abomasum of animals and crosses the intestine. In accordance with previous studies, our finding of *H*. *pylori* in milk might be attributed to contamination from feces^34–36,38^ or from the surrounding envirnoment^27^.

Irrespective of the species of animals or type of samples, all the 76 H. *pylori* PCR positive strains carried the virulence gene *vacA*, including 58 *cagA*^+^*vacA*^+^ and 18 *cagA*^−^*vacA*^+^ strains. The *vacA* allelic types, *s1a*, *s1b*, *s2*, *s1b m1*, *m2*, *i1*, *and i2* were detected, but the majority of the strains were of the *cagA*^+^*vacA*^+^*s1a m1 i1* genotype combination. This is consistent with the finding that *s1 m1 i1* predominates over *s2 m2 i2* in milk and meat from livestock^39^. Similar strains carrying *cagA*^+^*vacA*^+^*s1 m1* or *m2* were previously reported to be the most common genotypes among gastric cancer and gastritis patients in Egypt^40–42^, suggesting a relation between *H*. *pylori* isolated from livestock and humans. Furthermore, human studies worldwide have shown that *s1 m1 i1* strains are associated with severe gastric mucosal damage^21,43^, whereas, *cagA* gene contributes to induction of oncogenic signaling in gastric epithelial cells^19,21^. It is noteworthy to mention that our study does not exclude the co-presence of non-pylori *Helicobacter* species in the livestock and further work is required to examine their presence using species specific-primers^44^.

The 76 *H*. *pylori* PCR positive samples were further subjected to bacterial culture, 13 samples were culturable and showed the presence of the spiral viable form (SVCF) of *H*. *pylori* (Fig. 2). The negative bacterial culture results of some of the PCR positive samples are not due to technical error, as the positive control was culturable and the PCR negative samples were non-culturable. Additionally, there was no association between the non-culturability of the PCR positive samples and the type of samples (milk and feces) or the species of animals. Since the PCR-positive-bacterial-culture-negative samples carried the *H*. *pylori* virulence factors *vacA* and *cagA*^12,45,46^, we concluded that those samples might contain a non-culturable form of *H*. *pylori*. In this regard, previous studies^12,13,15,46,47^ reported the existence of *H*. *pylori* in a non-culturable form, which cannot be detected by the bacterial culture, but rather by using PCR^12,13,15^.

Almost all (n = 12) of the 13 SVCF isolates harbored the *cagA*^+^*vacA*^+^*s1a m1 i1* genotype, which is consistent with the genotype of the PCR positive strains. Sequencing of the 13 SVCF isolates based on Helicobacter-genus 16s rRNA confirmed that they are all *H*. *pylori* and excluded the presence of non-pylori Helicobacter species. Nevertheless, the 13 SVCF isolates were from different animal species, they were genetically identical (Fig. 3), which agrees with Yamoaka^37^ that *H*. *pylori* strains from the same geographical regions are akin. Unfortunately, we could not find any sequence of *H*. *pylori* isolated from Egypt in the NCBI gene bank to further support this explanation. Moreover, since none of the previous *H*. *pylori* isolates from livestock^24,33–36^ have been sequenced, we cannot determine the extent of similarity between our strains and other animal isolates. Interestingly, our isolates showed genetic relatedness to *H*. *pylori* strains from duodenal ulcer and gastritis patients in Asia (Fig. 3). This points to the presence of highly virulent *H*. *pylori* strains in apparently healthy livestock similar to those common in humans, elucidating that animals can act as silent spreaders of the bacterium. Our findings are in accordance with other studies that showed high prevalence of *H*. *pylori* in humans having contact with animals^6,32,33^ and their family members^31^.

The present study showed that feces of livestock represent the main shedding site of *H*. *pylori* and that the genotypes of fecal and milk strains from the same animal are identical, suggesting fecal contamination of milk^34,36,38^. This can happen due to lack of hygiene measures^36^ during milking like improper cleaning of the teats from residues of feces, allowing direct contact between milk and feces of the same animal, or indirectly from hands, clothes or boots of human milkers contaminated with animal fecal matter. Nowadays, the people prefer the heat processed milk, particularly the UHT over the raw milk, because of their safety and the long shelf life time. It is unlikely that *H*. *pylori* can survive the Ultra Heat Treatment, whereas, contamination of the UHT milk could happen either during production or post-processing due to poor hygienic handling of the product^27^. Therefore, the capability of *H*. *pylori* fecal isolates (SVCF) to survive in UHT milk under different temperature and time period was examined. We found that the SVCF of *H*. *pylori* survived in contaminated UHT milk for up to ten days at 4 °C, five days at 37 °C and one day at 40 °C. Our findings are in accordance with studies that demonstrated the survival of *H*. *pylori* for ten days in contaminated UHT milk^28^ and heat treated milk^25^ stored at 4 °C. Additionally, in the present study, the UHT milk stored at 4 °C showed a 3 log reduction in the number of *H*. *pylori* from day 0 (10^7^ cells/ml) to day10 (10^4^ cells/ml) following contamination, which is similar to that reported in the other two studies (3–4 log)^25,28^. Similarly, storage at 37 °C resulted in 3 log reduction (from 10^7^ cells/ml to 10^4^ cells/ml) in the number of *H*. *pylori*, but in five days. This agrees with Fan *et al*.^25^ who showed a similar log reduction between milk kept at 4 °C and that kept at 25 °C, despite shorter survival at 25 °C than at 4 °C. Furthermore, we demonstrated that the decrease in the SVCF coincides with the appearance of the coccoid viable non-culturable form (CVNCF), which survived in the UHT milk for up to 30 days. The genotype of the SVCF and CVNCF strains (*cagA*^+^*vacA*^+^*s1a m1 i1*) survived in milk was the same as the inoculated one, suggesting the conversion of the SVCF to a CVNCF. This agrees with the studies^13,48^ that showed transformation of the spiral form to the coccoid form under adverse conditions. Interestingly, our results revealed that the virulent *H*. *pylori* fecal strains can survive in contaminated UHT milk for up to 10 days in a number (10^4^ cells/ml) that can cause human infection^49^, which poses great public health hazard. We next fed healthy mice groups with the contaminated UHT milk containing the survived SVCF or CVNCF strains. Both strains were able to colonize the mice gastric mucosa in a similar level as the positive reference SS1 strain, providing an evidence for their infectivity. Indeed, the strains recovered from the gastric mucosa following infection were cytotoxic and carried the same genotype *cagA*^+^*vacA s1a m1 i1* as the inoculated strains, which excludes that the colonization might have been caused by strains other than the inoculated one. An important finding was the culturability of the strains isolated from the CVNCF mice group. This indicates a reversion of the inoculated CVNCF to a SVCF following gastric infection, which agrees with previous studies^48,50^. Collectively, our experimental findings demonstrate the viability of the CVNCF that was formed in the UHT milk contaminated with the SVCF and the ability to revert to its original spiral form when conditions are appropriate, causing infection. Another possible explanation for the gastric infection of the CVNCF group could be that the UHT milk that was given to the CVNCF mice group might have contained few numbers of SVCF which was not detectable by hemocytometer or the bacterial culture and have increased upon infection. However, the hemocytometer reading was confirmed by the bacterial culture method which has a sensitivity greater than 90%^51^, this explanation cannot be excluded.

In conclusion, the present study demonstrates the occurrence of virulent *H*. *pylori* strains in milk and feces from apparently healthy cattle, buffaloes and sheep in Egypt. *CagA*^+^*vacA s1a m1 i1* was the most common genotype, which was previously shown to be associated with human gastric cancer and gastritis in Egypt. Feces represent the main shedding site of *H*. *pylori*, suggesting feces as a possible source of milk contamination. Moreover, the fecal strains can persist in contaminated UHT milk by going through a viable non-culturable state which can revert back to a cytotoxic virulent culturable form under suitable conditions and induces infection. This represents a great public health risk as apparently healthy animals can silently spread virulent infectious strains of *H*. *pylori* to humans and to the environment. Considering that *H*. *pylori* is unlikely to survive the pasteurization or UHT process and given that post-processing contamination can happen, the more likely source of infection would be the raw milk.

## Materials and Methods

The experimental design of the current study is summarized in Fig. 1.

### Samples

Samples were collected from apparently healthy farm animals, they were all of the native Egyptian breed and were reared in a semi-intensive system. They include 70 cows (mean age 2.5, range 2–3 years), 60 domestic buffaloes (mean age 2.8, range 2–3.8 years), and 50 sheep (mean age 7, range 6–8 months). The animals were derived from three governorates in Egypt, which are known to have a high density of animals (EL-Giza, EL-Fayom, and EL-Qalyubia. Milk and feces were taken from each animal during the period from July to December 2015.

The freshly voided fecal samples were collected in sterile plastic cups, whereas the milk samples were taken in sterile falcon tubes, both fecal and milk samples were transported in ice chest. Each sample was divided into 2 parts, one used for PCR and the other for bacterial culture. All samples were then kept at −80 °C until used.

### Ethics approval

Protocols for sample collection as well as the experiment plan, including using lab mice were performed according to the guidelines of the Cairo University Council on Animal Care and were approved by the Scientific Research Committee and Bioethics Board of Cairo University, Faculty of Veterinary Medicine, Cairo, Egypt.

### Molecular identification

DNAs were extracted from fecal and milk samples using QIAamp tissue kit and QIAamp blood mini kit (Qiagen, Hombrechtikon, ZH, Switzerland), respectively, according to the manufacturer’s instructions with some modifications^52,53^.

#### Detection of *Helicobacter pylori*

Nested PCR was performed to amplify *H*. *pylori*-specific 16s rRNA gene using three oligonucleotide primers^54^ Hp1, Hp2, and Hp3 (Table 1). As previously described, this method increases sensitivity and specificity for detection of *H*. *pylori* and reduces the risk of cross reaction with non-pylori Helicobacter species^54–56^. A conventional PCR was also accomplished to amplify *H*. *pylori ureA* gene using primers^57^ listed in Table 1.

#### Determination of the virulence genes *vacA* and *cagA PAI*

Conventional PCR to amplify *cagA PAI* and *vacA* genes was run for samples positive for *H*. *pylori*-specific 16s and *ureA* genes, using primer sets^58,59^ described in Table 1. Empty-site PCR^60^ was used to confirm the absence of *cag PAI* in the negative strains (Table 1). The *cag PAI* is located between the two proteins coding *H*. *pylori* genes Hp0519 and Hp0549. The empty-site forward primer binds to Hp0519, and the reverse primer binds to Hp0549. Since the size of *cag PAI* (40 kb) is too large to be amplified by PCR, only *cag PAI* negative strains yield a product of 550 bp that represents the empty site.

#### Reagents and amplification conditions of the conventional and nested PCR

The sequences of all primers used and PCR conditions are listed in Table 1. Negative control (PCR mixture with nuclease free water instead of the template DNA) and 50 ng positive control (DNA from *H*. *pylori* reference strain SS1) was included. The Dream Taq Green PCR Master Mix (2X) and the nuclease free water were purchased from Thermo Fisher Scientific (Waltham, MA USA). The nested and conventional PCR amplifications were performed in the Swift MiniPro Thermal Cycler (ESCO Technologies Inc., Pennsylvania, USA).

#### Analysis of PCR products

The PCR products were run on 1.5% agarose gel containing 0.5x TBE at 70 volts for 60 min and visualized using a UV transilluminator (IN Geniuse 3, Syngene, MD, USA) connected with Quantity-one 4.6.2 software image for capture and analysis. DNA ladder marker was run simultaneously, the O’RangeRuler100 bp DNA ladder (Size range: 100–1,500 bp; Thermo Scientific) was used for sizing all the tested genes except for the ureA gene, where the 100 bp DNA ladder (Jena Bioscience GmbH, Jena, Germany) with a size range of 100–1,000 bp was included.

### Isolation and identification of *Helicobacter Pylori*

Five grams of fecal samples were homogenized separately with 15 ml Brucella broth (Difco, New Jersey, USA) containing 20% glycerol (Sigma-Aldrich, St. Louis, MO, USA) and 0.5 g cholestyramine (Sigma-Aldrich). Two ml from each of the milk samples were inoculated in eight ml of Brucella broth. As a control, randomly selected negative PCR fecal (n = 2) and milk (n = 2) samples were included with or without the addition of *H*. *pylori* reference SS1 strain (McFarland 5) to be used as positive and negative controls, respectively. The control samples were treated the same way as the tested samples.

A loop-full from the prepared homogenates was streaked onto selective Brain Heart Infusion agar plates (Difco) supplemented with 7% defibrinated horse blood (Sigma-Aldrich), and antibiotics (Sigma-Aldrich), including vancomycin (10 mg/L), amphotericin B (4 mg/L), trimethoprim (5 mg/L) and polymyxin (10 mg/L) according to Forbes *et al*.^61^. The inoculated plates were incubated under microaerophilic conditions at 37 °C for 3 to 5 days. Suspected colonies were purified through subculture on Brain Heart infusion, then onto Brucella broth supplemented with 5% newborn calf serum. The pure colonies were subjected to microscopic examination, gram staining, and traditional biochemical tests^61^ including Urea hydrolysis, Catalase production, Oxidase production, Nitrate reduction, Glycine utilization, and Salt tolerance.

### Sequencing of *Helicobacter pylori* culture positive isolates

In order to confirm the identity of the *H*. *pylori* isolates, DNAs were extracted from the purified colonies of all isolates using QIAamp mini kit (Qiagen) and were amplified for *Helicobacter* genus-specific 16s using *C97* and *C05* primers^62^ (Table 1). The PCR products were purified using Qiaquick purification kit (Qiagen) and were sequenced using Big Dye Terminator V3.1 sequencing kit (Applied Biosystems, Waltham, MA, USA). The obtained nucleotide sequences were compared with those in the Public Database using the NCBI-BLAST server and were deposited in the GeneBank.

#### Phylogenetic analysis

The obtained nucleotide sequences were compared with those available in public domains using the NCBI-BLAST server. Sequences were downloaded and imported into the BioEdit program version 7.0.1.4 for multiple alignments using the BioEdit Clustal W program. Phylogenetic analysis was performed with the MEGA program version 7 using the neighbor-joining approach.

### Cytotoxicity of *Helicobacter pylori* isolates using vacuolization assay in vero cells

A total of 2.1 × 10^6^ vero cells were seeded into a 75 cm^2^ tissue culture flask in Earle’s minimum essential medium (Gibco) and were incubated for 24–48 hours to produce a confluent monolayer of 8.4 × 10^6^ according to Tee *et al*.^63^. The supernatant was then replaced by 25 ml fresh medium containing 5 ml broth culture filtrate of *H*. *pylori* test strains and vero cells were incubated at 35 °C in the presence of CO_2_. The intracellular vacuolization of vero cells was examined after 24 and 48 hours with an inverted microscope and used as an indicator of cytotoxicity of *H*. *pylori* strains.

### Experimental contamination of ultra-high temperature (UHT) milk with *Helicobacter pylori* spiral viable culturable form (SVCF) isolated from feces of livestock

To exclude any variable factors which might influence the results, one liter bottle of UHT cow milk obtained from the market was divided into 126 portions, each with 5 ml. The 126 portions were used to examine in duplicates one test strain, one positive strain, and negative control, at three different temperatures (4, 37, and 40 °C) for seven time points (days 1, 2, 3, 5, 10, 15, and 30). The bacterial strains used were the test *H*. *pylori* SVCF strain (cytotoxic *cagA*^+^ *vacA s1a m1 i1* isolated from cow fecal sample) and the SS1 reference strain as positive control. The strains were cultured as mentioned above in section Isolation and identification of *Helicobacter pylori*, each whole culture was harvested, re-suspended in 23 ml sterile Phosphate-buffered saline (PBS; Lonza, Basel, Switzerland). Each 5 ml UHT milk portion was inoculated separately with 0.5 ml of the prepared suspension of the test *H*. *pylori* SVCF strain, SS1 reference strain (positive control) to reach a concentration of 1.5 × 10^7^ cells/ml milk (as measured by the standard plate count method)^29^, or left uninfected (negative control). The concentration at day 0 was further examined using hemocytometer, and all portions were then incubated at its particular temperature and time point. At the end of each time point, the incubated UHT milk portions were serially diluted in PBS, the SVCF and the coccoid viable non-culturable form CVNCF were counted using a hemocytometer. The milk portions were further investigated by PCR to detect both forms of *H*. *pylori* and by RT-PCR to detect mRNA expression of *cagA* and *vacA*. Bacterial culture and identification were also performed for all milk portions. It is important to note that the milk bottle was tested before the experimental inoculation, for the absence of *H*. *pylori* as well as other *Helicobacter species* using *Helicobacter* genus**-**specific 16s PCR.

#### Criteria used to distinguish between the spiral viable culturable (SVCF) and coccoid viable non-culturable form (CVNCF)

Under electron microscope, the SVCF is a spiral rod shape bacilli while, the CVNCF has a spherical coccoid shape^12,15^. Briefly, each milk sample was centrifuged at 13,000 rpm for 10 min., the supernatant was then removed, and the bacterial sediment was resuspended in 10 ul PBS. The bacterial suspension was bound to 300-mesh Cu-coated grids (Sciences Services GmbH, München, Germany) and stained with 2% uranyl acetate. The grid was dried for 12 hours and analyzed under JEM-1200 EX II transmission electron microscope (JEOL, Tokyo, Japan). This was performed at the Electron Microscope Unit, Military Veterinary Hospital, Nasr City, Cairo, Egypt. Another criteria, the SVCF and the CVNCF forms can be detected by PCR, but only the SVCF can be isolated using bacterial culture^13,19^.

### Experimental infection of Balb/c mice with the artificially contaminated UHT milk containing either the survived *Helicobacter pylori* forms

Prior to the experimental infection, the Helicobacter status of the Balb/c mice included in the study, was examined by *Helicobacter* genus-specific 16s PCR on DNA extracted from the mice fecal samples using QIAamp tissue kit (Qiagen). The fecal samples were collected twice, the first was upon arrival of the mice and the second at the same day of the experiment before inoculation. The UHT milk portions included in this experiment were verified to contain only one form of *H*. *pylori* (SVCF or CVNCF) by subjecting them to electron microscope, bacterial culture and PCR.

According to previous studies^28,64,65^, the concentration of *H*. *pylori* and the period of infection used in our experiment were chosen.

Balb/c mice (n = 16) were divided equally into 4 groups. The negative control group received 0.4 ml uninfected UHT milk. The SVCF-Group and the CVNCF-Group were given 0.4 ml of the artificially contaminated UHT milk suspensions containing 10^9^ cfu/L SVCF or CVNCF forms, respectively. The Positive control group was given UHT milk containing SS1 reference strain. The four groups received the treatment orally using a syringe for 40 days with 3-day intervals. Each mouse received 14 doses starting at day one with the last dose was at day 40. This was followed by fasting the animals with free access to water until they were sacrificed one day after the last dose. After being sacrificed, their stomachs were removed, opened, and washed with sterile saline. The 3 × 3 mm sized tissue fragments were taken from the pylorus part of one-third of the gastric mucosa and were homogenized. The gastric mucosa homogenates were examined for the *H*. *pylori* load using real-time/quantitative PCR (qPCR) and for mRNA expression of *cagA* and *vacA* by reverse transcription PCR (RT-PCR) as described below in the next two sections, respectively. The homogenates were further subjected to bacterial culture and the cytotoxicity of the isolated strains was determined using a vacuolization assay in vero cells.

### Assessment of Helicobacter *pylori* load in mice gastric mucosa using real-time/quantitative polymerase chain reaction (qPCR)

Twenty five gram of gastric sample from each mouse was homogenized and 200 ul of each homogenate was used for DNA extraction by using QIAamp tissue kit (Qiagen) according to the manufacturer’s instructions. The extracted DNAs were quantified using Qubit^®^ 2.0 Fluorometer (Thermo Fisher Scientific) and were subjected to qPCR^66,67^ targeting *H*. *pylori* species-specific antigen (*SSA*) gene using specific primers (Table 1). The qPCR reaction mixture for a total volume of 50 μl consisted of 1 μl of extracted DNA (200 ng), 12 μl (50 nM) of each primer, and 25 μl SYBR Green dye I master mix (Qiagen). All samples were amplified in triplicate and the reaction was run in the Gene Amp 5700 Sequence Detection System (Applied Biosystem) under the following conditions: preliminary denaturation at 95 °C for 5 min, followed by 40 cycles of denaturation at 95 °C for 30s, annealing at 65 °C for 30s, and primer extension at 72 °C for 30s. The readout of the assay is represented by cycle threshold (Ct) which is the point at which the fluorescence rises above the background level. The *SSA* expression by all samples was normalized against the mouse housekeeping gene *GAPDH*. As a positive control, DNA was extracted from a pure culture of *H*. *pylori* SS1 reference strain and was subjected to six 10-fold serial dilution containing 10^1^−10^6^ fg bacteria (10 fg corresponds to 5 bacterial cells per 200 ng of host’s DNA)^66,67^. Each dilution was added to 200 ng genomic DNA extracted from uninfected gastric mucosa and was amplified in parallel with the samples. The standard curve was generated by plotting the amount of bacterial DNA in the dilution series against the corresponding Ct cycle number, (Fig. S5). The concentrations of *H*. *Pylori* DNA in the tested samples was determined by interpolation of the corresponding Ct value from the standard curve. The bacterial load is expressed as cell numbers per 200 ng mouse genomic DNA.

### Detection of mRNA expression of *vacA* and *cagA* by reverse transcription polymerase chain reaction (RT-PCR)

RNA was extracted from the UHT milk samples and mouse gastric mucosa using the RNeasy mini kit (Qiagen) with DNase treatment step using RNase-free DNase set (Qiagen). The concentration of the extracted RNA was measured by Qubit^®^ 2.0 Fluorometer (Thermo Fisher Scientific). Reverse transcription (RT) and Uniplex-PCR targeting *cagA* and *vacA* alleles were carried out in a single step using OneStep RT-PCR Kit (Qiagen). The extracted RNA (1 μg) was mixed with the RT-PCR kit reagents according to the recommendation of the supplier. The primers targeting *cagA*, *vacA* alleles, and *H*. *pylori*-specific 16s rRNA genes (Table 1) were added separately to the reaction mixture. The *H*. *pylori*-specific 16s rRNA was used as a control to exclude the presence of PCR inhibitors in the samples. The RT-PCR reactions were cycled in the Swift MiniPro Thermal Cycler (ESCO Technologies Inc.) under two steps. The first step is the cDNA synthesis by incubation at 70 °C for 50 min, then at 94 °C for 2 min; the second step is amplification of the synthesized cDNA through 10 PCR cycles of denaturation at 94 °C for 30s, annealing at 60 °C for 30s, and extension at 70 °C for 1 min. This was followed by 25 cycles of denaturation at 94 °C for 1 min, annealing at 60 °C for 1 min, and extension at 70 °C for 1 min with a final extension of 7 min at 70 °C. The RT-PCR products were detected by 1.5% agarose gel electrophoresis containing 0.5x TBE at 70 volts for 60 min and visualized using a UV transilluminator (IN Geniuse 3, Syngene, MD, USA) connected with Quantity-one 4.6.2 software image for capture and analysis. The 100 bp DNA ladder marker (Size range: 100–1,000 bp, Jena Bioscience GmbH) was run simultaneously.

### Statistical analysis

NCSS 9 software (NCSS, Kaysville, UT) was used for statistical analyses. A chi-square test was used to determine whether there was a difference in the occurrence of *H*. *pylori* and animal species (Table 2). McNemar test was used to compare the occurrence of *H*. *pylori* between milk and fecal samples (Table 2). Repeated Measures ANOVA was used to examine the influence as well as the interaction of the factors, temperature [4 °C, 37 °C, and 40 °C] and time periods of storage [1–30 days] on the count of the SVCF and CVNCF of *H*. *pylori* (Fig. 5). On each occasion, P values ≤ 0.05 were regarded as significant.

## Electronic supplementary material

Fig.S1, S2, S3, S4, S5, and their legends

## Data Availability

All data generated or analyzed during this study are included in this published article (and its Supplementary Information Files).
