# Supplementary material for: Occurrence of cagA+vacA s1a m1 i1 Helicobacter pylori in farm animals in Egypt and ability to survive in experimentally contaminated UHT milk
Source: Sci Rep. 2018 Sep 24;8:14260. doi: 10.1038/s41598-018-32671-0 (PMC6155285; doi:10.1038/s41598-018-32671-0)
Supplement: Supplementary file 1 — Fig.S1, S2, S3, S4, S5, and their legends [file 41598_2018_32671_MOESM1_ESM.pdf]

Running Title: Survival of non-culturable *H. pylori* in contaminated UHT milk

Occurrence of *cagA*$^{+}$ *vacA s1a m1 i1 Helicobacter pylori* in farm animals in Egypt and ability to survive in experimentally contaminated UHT milk

Mahmoud Elhariri[1], Dalia Hamza[2], Rehab Elhelw[1], Eman Hamza[2]*

[1]Department of Microbiology and Immunology, Faculty of Veterinary Medicine, Cairo University, Egypt.

[2]Department of Zoonoses, Faculty of Veterinary Medicine, Cairo University, Egypt.

***Correspondence**: Dr. Eman Hamza, Department of Zoonoses, Faculty of Veterinary Medicine, University of Cairo. Giza square, PO Box 12211 Cairo, Egypt

Tel. [+2] 01020484953, [+41] 0788700567
Fax. [+2] (02) 35725240
Email: e.hamza@gmx.ch

Keywords: Helicobacter pylori, coccoid form, spiral form, viable culturable H. pylori form, viable non-culturable H. pylori form, milk, feces

Fig. S1: Agarose gel electrophoresis of PCR products of DNA extracted from milk and fecal samples collected from cows, buffaloes and sheep.
A. The extracted DNA was amplified for *H. pylori*-specific-16s rRNA using Hp1, Hp2 and Hp3 oligonucleotides primers. Lane 1: Marker, O`RangeRuler 100bp DNA ladder (size range: 100-1,500 bp, Thermo Fischer). **Lane 2:** positive control *H. pylori*-specific-16s rRNA gene at 109 bp, **Lane 3:** negative control. **Lane 4:** Cow fecal sample. **Lane 5:** Buffalo fecal sample. **Lane 6:** Sheep fecal sample. **Lane 7:** Cow milk sample. **Lane 8:** Buffalo milk sample. **Lane 9:** Sheep milk sample. **Lane 10:** Cow fecal sample.

B. Amplification of the extracted DNA for detection of *ureA* gene. Lane 1: 100bp DNA ladder (size range: 100-1,000 bp, Jena Bioscience GmbH). **Lane 2:** positive control *ureA* gene at 411 bp, **Lane 3:** negative control. **Lane 4:** Cow fecal sample. **Lane 5:** Buffalo fecal sample. **Lane 6:** Sheep fecal sample. **Lane 7:** Cow milk sample. **Lane 8:** Buffalo milk sample. **Lane 9:** Sheep milk sample. **Lane 10:** Cow fecal sample.

Fig. S2: PCR photos showing genotype of *cagA* and diverse alleles of *vacA* in DNA extracted from milk and fecal samples collected from cows, buffaloes and sheep.
A) *cagA* gene at 352 bp, **B)** *s1a vacA* gene at 190 bp, **C)** *m1 vacA* gene at 290 bp, **D)** *i1 vacA* gene at 426 bp. **M:** Marker, Marker, O`RangeRuler 100bp DNA ladder (size range: 100-1,500 bp, Thermo Fischer). **Lane 1:** Negative control**. Lane 2:** Cow fecal sample**. Lane 3:** Buffalo fecal sample. **Lane 4:** Sheep fecal sample. **Lane 5:** Cow milk sample. **Lane 6:** Buffalo milk sample. **Lane 7:** Sheep milk sample.

Fig. S3: PCR photos of *Helicobacter* genus-specific 16s in DNA extracted from UHT milk and fecal sample of the mice groups prior to the experimental infection.
M: Marker, O`RangeRuler 100bp DNA ladder (size range: 100-1,500 bp, Thermo Fischer). **Lane 1:** positive control of *Helicobacter* genus-specific 16s at 1200 bp, **Lane 2:** The UHT milk. **Lane 3:** Pooled DNA from the negative control mice group. **Lane 4:** Pooled DNA from the positive control mice group. **Lane 5:** Pooled DNA from the **SVCF** mice group. **Lane 6:** Pooled DNA from the **CVNCF** mice group.

Fig. S4: RT-PCR photos showing mRNA expression of *cagA* gene *(A)*, *s1a (B), m1 (C), i1(D)* alleles of *vacA gene* and *H. pylori*-16srRNA *(E)* in experimentally contaminated UHT milk (Lanes 3-5) and in gastric mucosa from experimentally infected balb/C mice (Lanes 1-2; 6-7).
M: Marker, Marker, O`RangeRuler 100bp DNA ladder (size range: 100-1,500 bp, Thermo Fischer). **Lane 1:** Negative control (Mouse from the group that was given uninfected UHT milk). **Lane 2:** Positive control (Mouse from the group that was given UHT milk containing SS1 reference strain). **Lane 3:** Contaminated UHT milk (inoculated with **SVCF** isolated from cow fecal sample) and incubated at 40°C for 5 days. **Lane 4:** Contaminated UHT milk incubated at 37°C for 10 days. **Lane 5:** Contaminated UHT milk incubated at 4°C for 5 days. **Lane 6:** Mouse from the group that was given pasteurized milk containing the coccoid **CVNCF** of *H. pylori*. **Lane 7:** Mouse from the group that was given **SVCF** of *H. pylori.*

Fig. S5: Standard curve for SSA amplification. Six 10-fold serial dilutions containing 10^1-10^6 fg of bacterial DNA were amplified in the presence of 200 ng of mouse genomic DNA by real-time PCR using SYBR Green I dye. 10 fg (corresponds to 5 bacterial cells) per 200 ng of host's DNA. The Cycle Threshold (C_T) values were plotted against input template DNA. Correlation coefficients obtained by linear regression analysis of several independent experiments were higher than $R^2 = 0.99$, indicating highly efficient and reproducible reactions.

Fig. S1

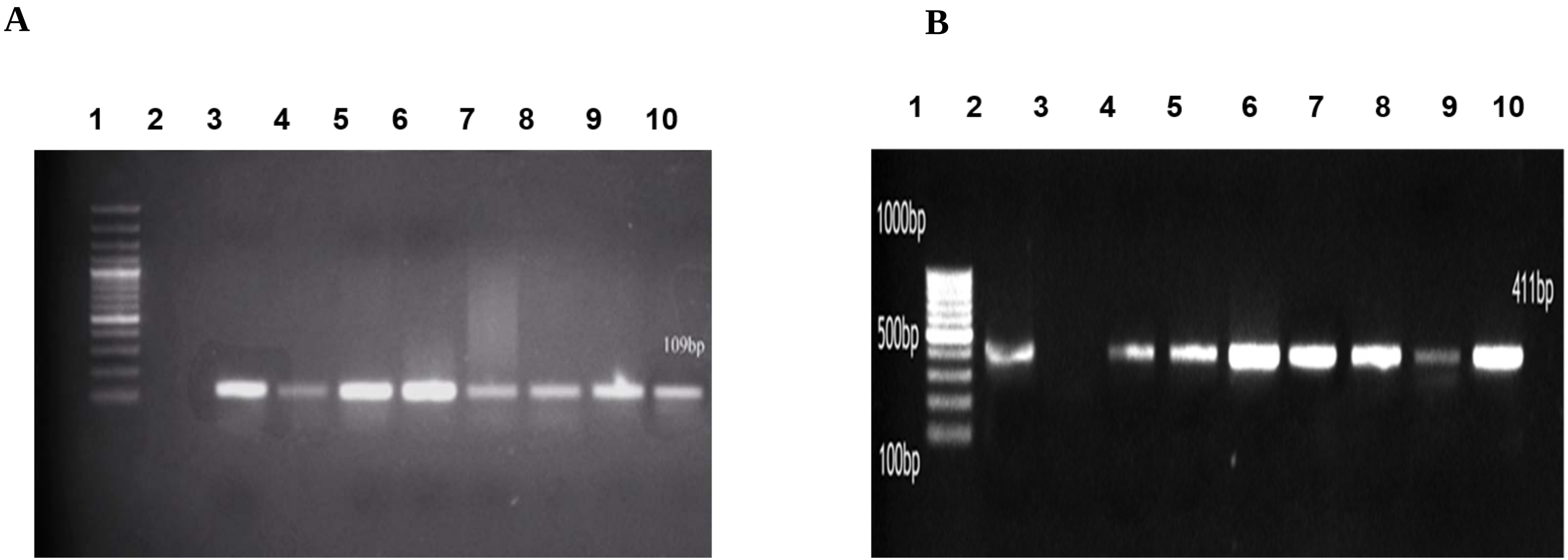
A
1 2 3 4 5 6 7 8 9 10
109bp
B
1 2 3 4 5 6 7 8 9 10
1000bp
500bp
100bp
411bp

Fig. S2

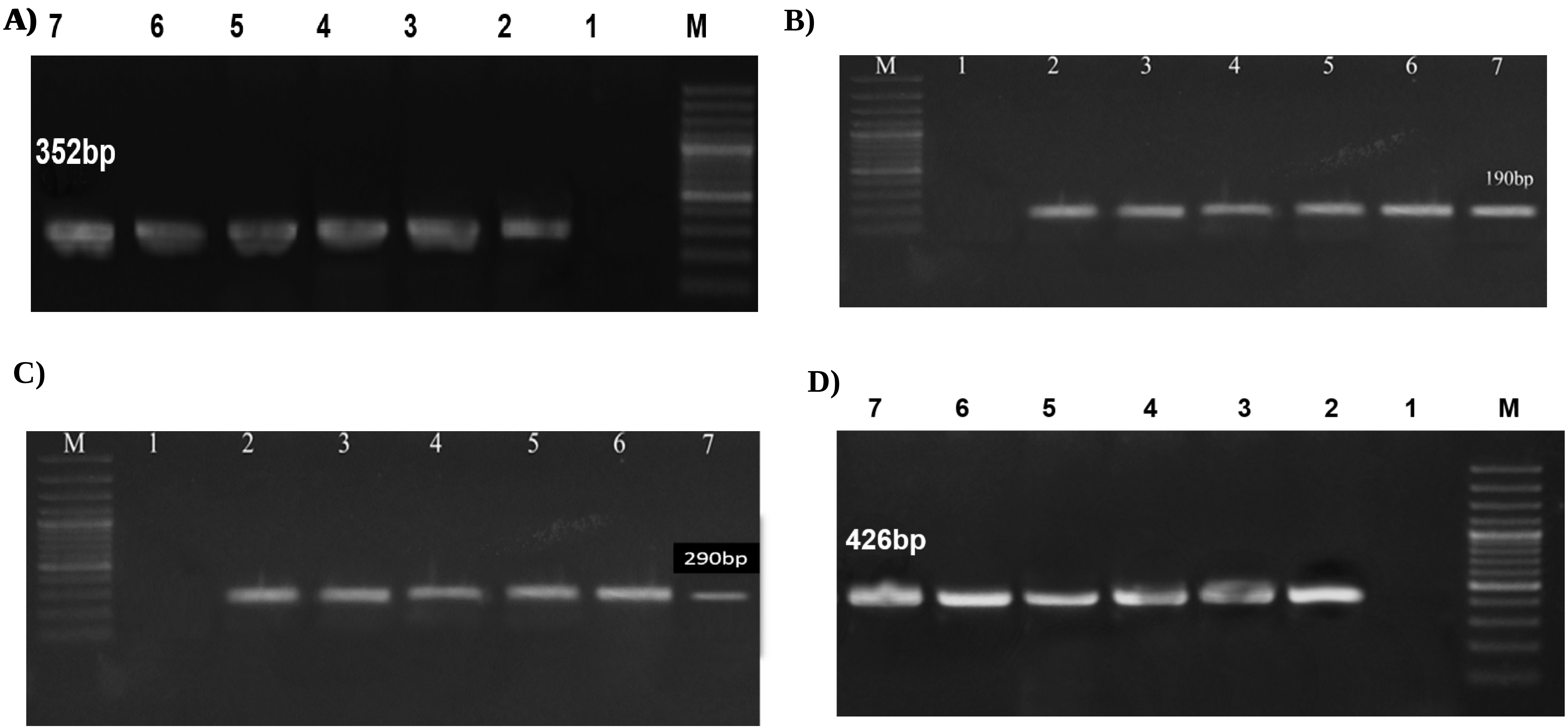

A)
7 6 5 4 3 2 1 M
352bp
B)
M 1 2 3 4 5 6 7
190bp
C)
M 1 2 3 4 5 6 7
290bp
D)
7 6 5 4 3 2 1 M
426bp

Fig. S3 ***Helicobacter genus-specific- 16s*** **DNA**

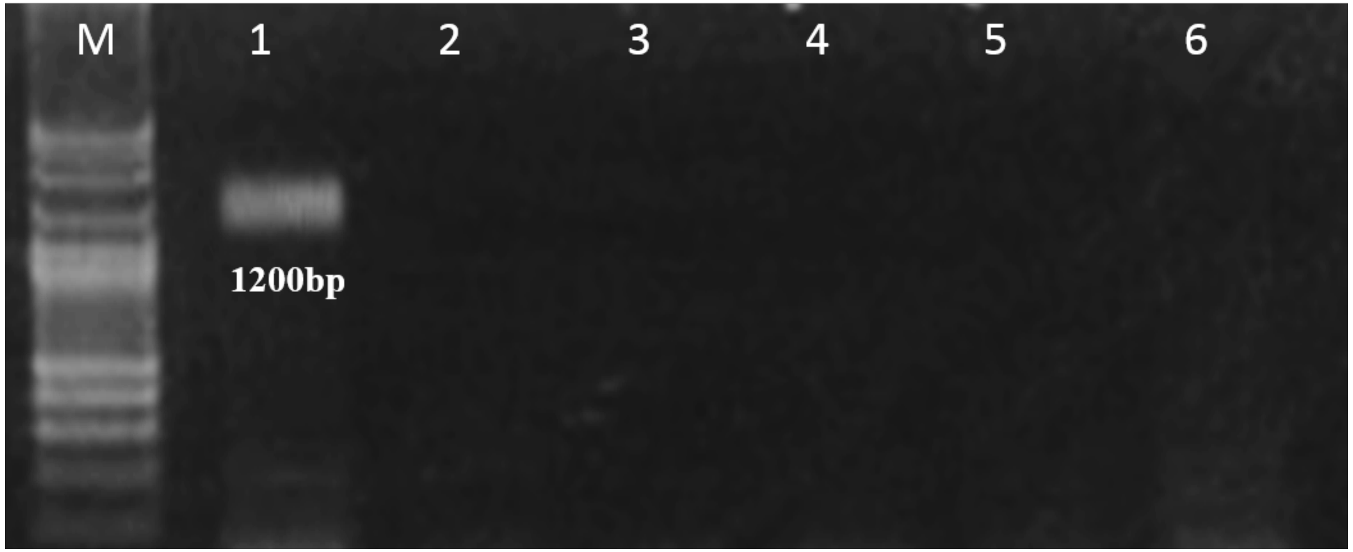

Fig. S4

A) *cagA* mRNA

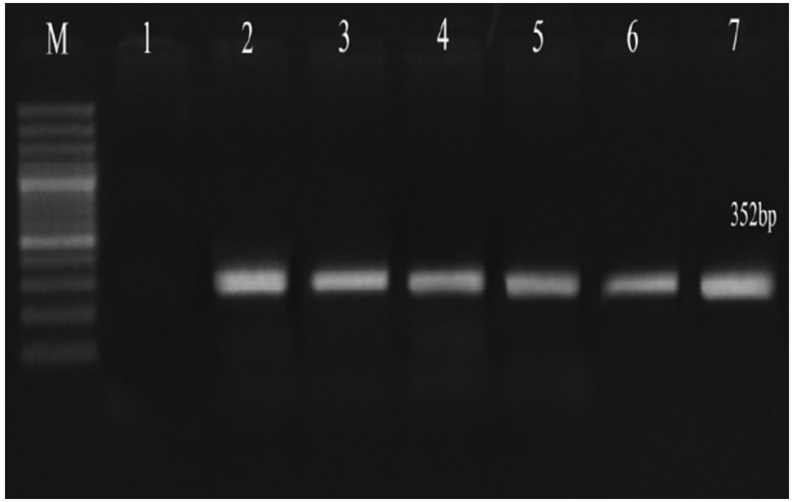

B) *vacA s1a* mRNA

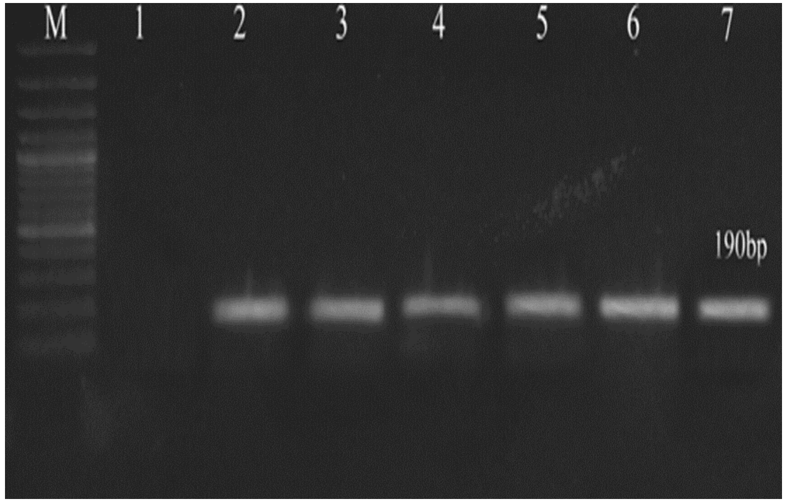

C) *vacA m1* mRNA

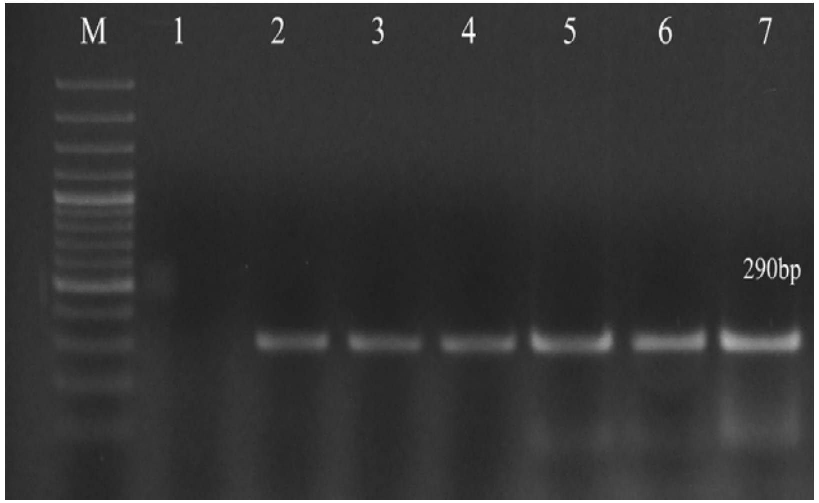

D) *vacA i1* mRNA

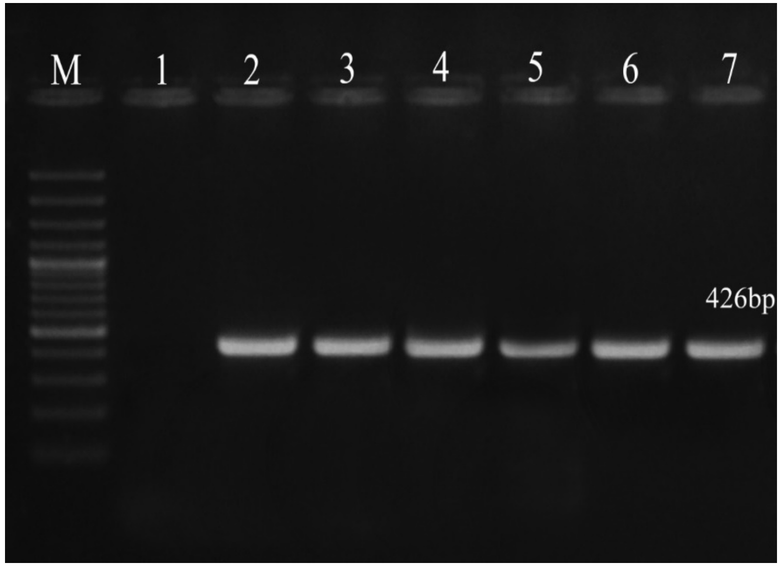

E) *H.pylori-specific 16s rRNA* mRNA

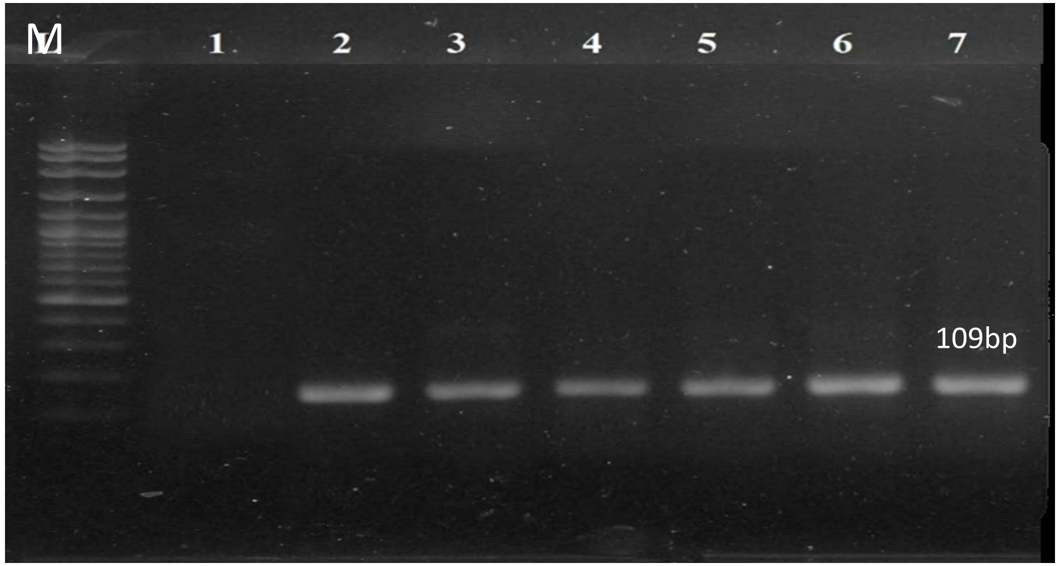

Fig. S5

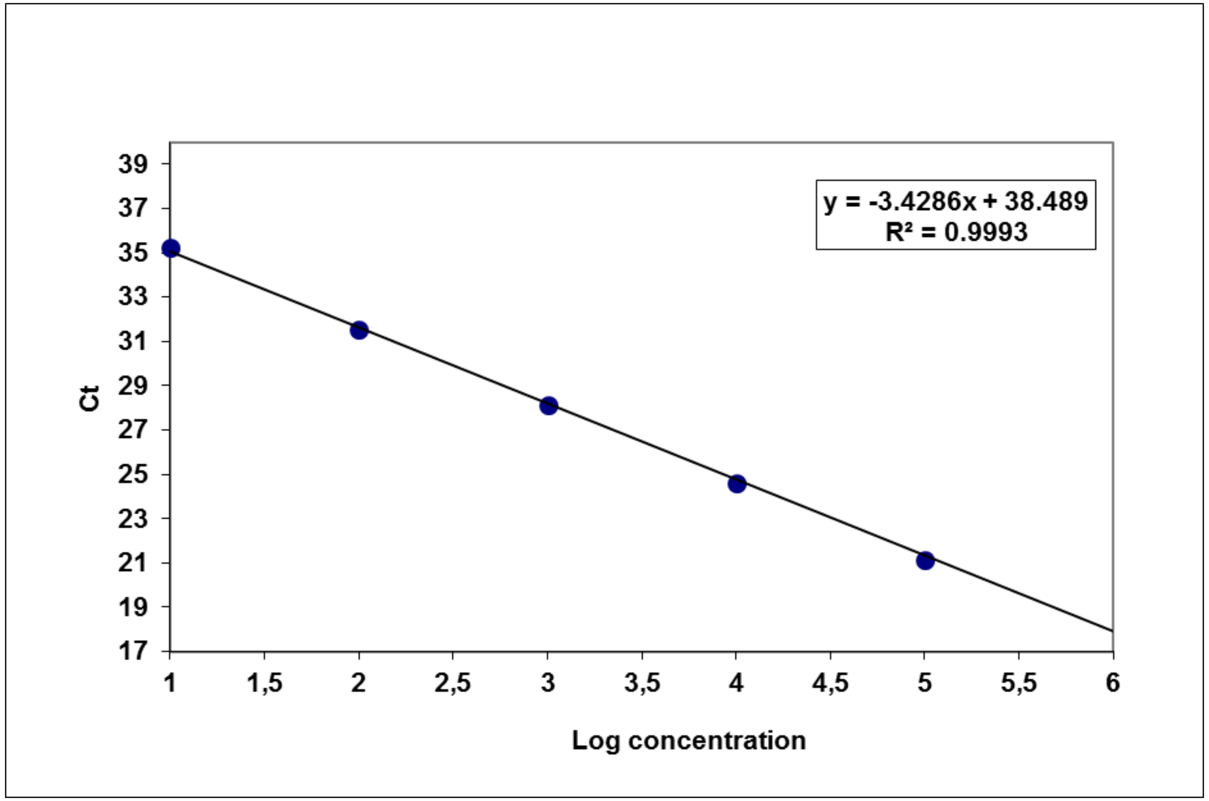

y = -3.4286x + 38.489
R² = 0.9993
Ct
39
37
35
33
31
29
27
25
23
21
19
17
1
1,5
2
2,5
3
3,5
4
4,5
5
5,5
6
Log concentration
